# Memtransistors Based on Nanopatterned Graphene Ferroelectric Field-Effect Transistors

**DOI:** 10.3390/nano10071404

**Published:** 2020-07-19

**Authors:** Mircea Dragoman, Adrian Dinescu, Florin Nastase, Daniela Dragoman

**Affiliations:** 1 National Institute for Research and Development in Microtechnology (IMT), Str. Erou Iancu Nicolae 126 A, 077191 Voluntari, Romania; adrian.dinescu@imt.ro (A.D.); florin.nastase@imt.ro (F.N.); 2 Physics Faculty, University of Bucharest, P.O. Box MG-11, 077125 Bucharest, Romania; daniela@solid.fizica.unibuc.ro; 3 Academy of Romanian Scientists, Str. Ilfov, Nr. 3, 050044 Bucharest, Romania

**Keywords:** graphene, HfO2-based ferroelectrics, memtransistors

## Abstract

The ultimate memristor, which acts as resistive memory and an artificial neural synapse, is made from a single atomic layer. In this manuscript, we present experimental evidence of the memristive properties of a nanopatterned ferroelectric graphene field-effect transistor (FET). The graphene FET has, as a channel, a graphene monolayer transferred onto an HfO_2_-based ferroelectric material, the channel being nanopatterned with an array of holes with a diameter of 20 nm.

## 1. Introduction

The ultimate memristor is based on a single atomic layer/a two-dimensional (2D) material and metallic electrodes. Significant progress towards such a memristor was made by using 2D transition metal dichalcogenides (TMDs) such as MoS_2_, which can work as microwave switches up to 50 GHz [1,2]. Many 2D materials show excellent memristive properties, with applications in crossbar arrays for neuromorphic computing [3,4].

The first atomristors were vertical metal–semiconductor–metal (MSM) structures, mimicking the original memristors based on very thin oxides with thicknesses of a few nanometers, which are known as valence change memristors (VCMs) or filament memristors. VCM memristors have top and bottom electrodes, made of the same or different metals, deposited on either side of a very thin oxide such as HfO_x_, TiO_x_, or a 2D material and other oxide types (see [3,4,5,6]).

By using few-atom-thick 2D materials such as MoS_2_, three-terminal lateral memristors termed as memtransistors were developed [7,8,9]. The memtransistors have important advantages over the two-terminal vertical memristors: (i) lack of electroforming process for the wake-up of conductive filaments, (ii) tunable memory functions and high on-off ratios, (iii) lack of sneak paths in crossbar arrays unlike in VCM memristors (the most important advantage) [10] and (iv) high mobility (i.e., 0.6 cm^2^/Vs) [8], nine orders of magnitude higher than in VCM memristors, where it is 10^−10^ cm^2^/Vs [11]. 

Graphene was the first 2D material used for resistive memories/memristors [12]. Graphene oxide is now being extensively used for the same purpose [13]. Taking into account the previous considerations on 2D memristors, this article demonstrates the possibility of producing memtransistors based on a graphene monolayer, which is a single-atom-thick carbon material. Ferroelectric transistors are very promising devices for random-access memories, neuromorphic applications using ferroelectric transistors as artificial neurons [14], and ferroelectric tunneling junctions as synapses since they act as memristors [15]. Recently, 2D ferroelectric transistors were fabricated using flakes of α-In_2_Se_3_, which is a semiconductor ferroelectric [16], but its memristive properties were not studied. Therefore, the memristive behavior of ferroelectric FETs is an important issue to be studied. 

In this respect, we fabricated field-effect transistors (FETs) at the wafer scale having, as a channel, a graphene monolayer nanopatterned with holes 20 nm in diameter and transferred over a ferroelectric 6 nm HfO_2_ substrate doped with Zr (denoted further as HfZrO), grown over 20 nm thick Al_2_O_3_ on Si. Both HfZrO and Al_2_O_3_ were grown by atomic layer deposition (ALD) in the same batch. Note that the graphene FET on a non-ferromagnetic substrate, with the channel nanopatterned with nanosized holes, has already shown both high mobility and on–off ratio. Depending on the length of the graphene channel (i.e., the number of nanoholes), the mobility of this transistor could be engineered from 1000 cm^2^/Vs up to 10,000 cm^2^/Vs [17]. 

## 2. Materials and Methods

The growth procedure of 6 nm HfZrO by ALD on an Al_2_O_3_/Si substrate and the structural characterization for the experimental demonstration of ferroelectricity in HfZrO (XRD, PFM tests) were described in [18] and the associated supplemental information, and thus are not repeated here. The present HfZrO/Al_2_O_3_/Si structure was grown on the same equipment as in [16] and under the same conditions. HfZrO was the ferroelectric layer, and Al_2_O_3_ was a buffer layer to prevent the transport of charges from Si substrate to HfZrO. Moreover, the roughness of Al_2_O_3_ was under 1 nm, so it was the ideal substrate to grow HfZrO by ALD.

Then, the graphene monolayer was transferred onto the 4 inch wafer of HfZrO/Al_2_O_3_/Si by Graphenea, a high-quality graphene production company. In the following, we show that the presence of ferroelectricity in the graphene FET can also be deduced from DC measurements, that is, the drain current–top gate voltage hysteretic dependence, which is the imprint of any ferroelectric transistor.

**Figure 1:**
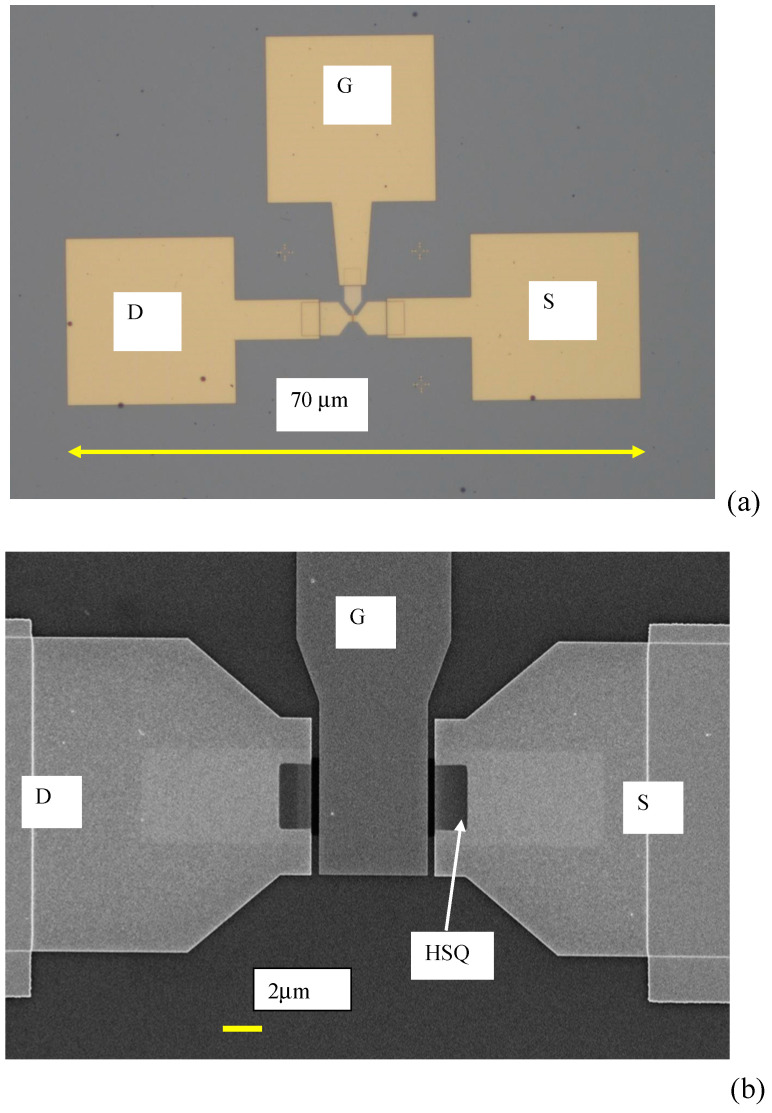
(**a**) Optical image, and (**b**) SEM image of the HfZrO/Al_2_O_3_/Si graphene field-effect transistor (FET).

**Figure 2:**
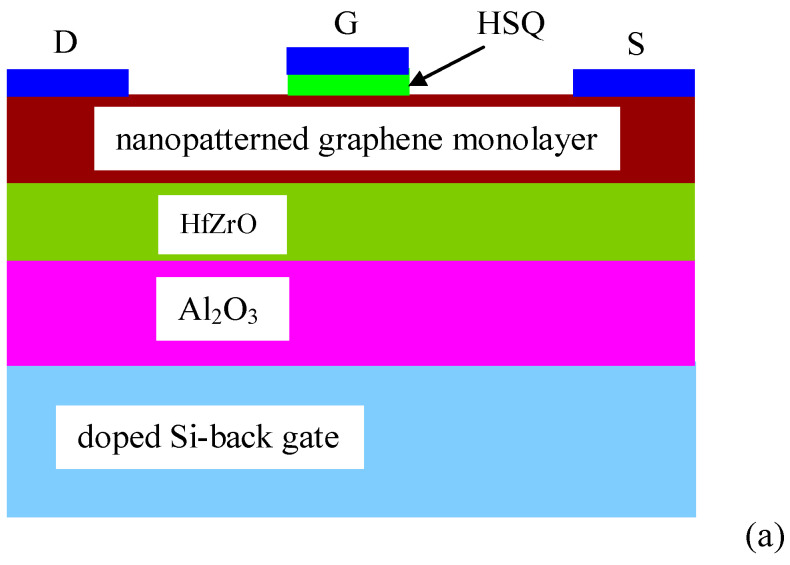

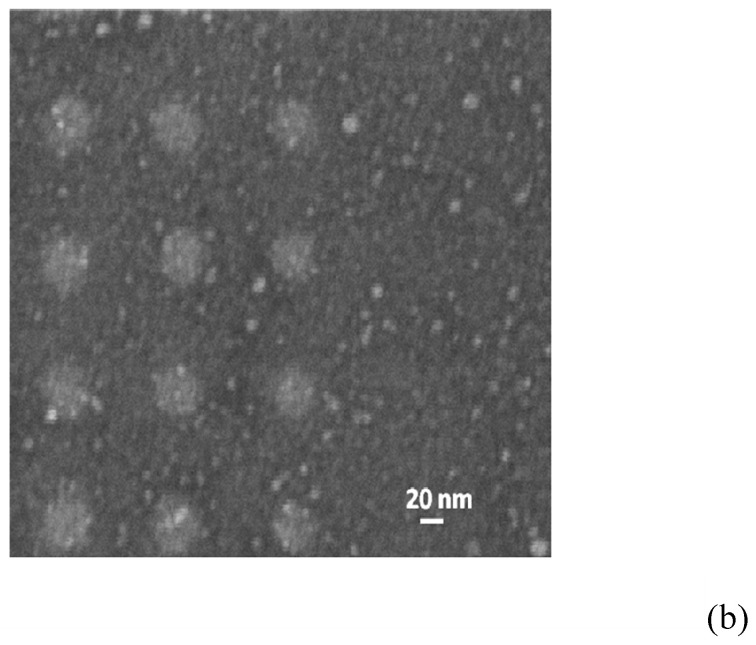
(**a**) Schematic cross section of the ferroelectric nanopatterned FET channel and (**b**) SEM image of the end section of graphene channel.

The fabrication of the FET was performed in several steps: (i) the graphene channel was patterned using the e-beam technique, with nanoholes 20 nm in diameter arranged in a square lattice with a periodicity of 100 nm (the e-beam technique for the patterning of nanoholes was recently described in detail in [17] and [19]), (ii) the patterned graphene channel was cut using oxygen plasma, (iii) source (S) and (D) drain contacts were deposited, (iv) the gate insulator, HSQ (Hydrogen Silsesquioxane), 30 nm in thickness was deposited and (v) the gate electrode was deposited. The FET electrodes were Ti/Au (5/35 nm) and were deposited by e-beam evaporation (TEMESCAL FC2000). Figure 1a shows the optical image of the transistor displayed on a chip with a channel length of 8 μm, and Figure 1b illustrates the SEM image of the transistor. In Figure 1b, the two rectangular shadows around the dark HSQ, which are brighter than the metal electrodes and denoted by D and S, represent the graphene channel. Figure 2a represents a schematic cross section of the nanopatterned ferroelectric graphene FET with its back and top gates, while Figure 2b shows an SEM photo of the end region of the graphene channel, indicating the nanopatterned holes.

## 3. Results and Discussion

The electrical characterization of graphene FETs at room temperature was performed using a Keithley SCS 4200 station, where the probe station for on-chip measurement was placed inside a Faraday cage, and the outputs of the DC probes were connected outside the cage, with electromagnetic shielded cables, to the low-noise amplifiers at the station. No fitting algorithms were used during or after measurements. Out of the 52 measured transistors, 16 did not work well due to a lack of adherence to the metallic contacts and cracks in the graphene channels. The remaining transistors showed similar results within an average 7–8% error. 

**Figure 3:**
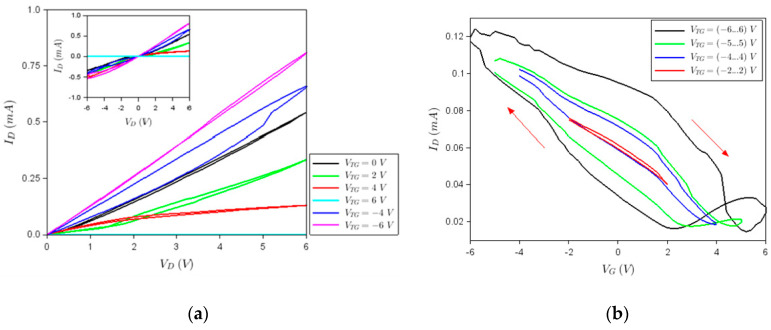
(**a**) Drain current versus positive drain voltages at various top gate voltages indicated in the legend when $V_{BG}$ = 0. Inset: full $I_{D}-V_{D}$ dependence for both drain voltage polarities. (**b**) Drain current versus top gate voltage hysteretic dependence at a different $V_{TG}$ sweep, when $V_{BG}$ = 0.

Figure 3a shows the dependence of the drain current ($I_{D}$) versus positive drain voltages ($V_{D}$) at various top gate voltages ($V_{TG}$) when the back gate voltage ($V_{BG}$) is zero, the inset illustrates the same dependence for both $V_{D}$ polarizations. It follows that when positive top gate voltages are applied progressively, the drain current decreases until the transistor reaches the OFF state at $V_{TG}$ = 6 V when the current is 10 nA (it cannot be distinguished from the horizontal axis). On the contrary, $I_{D}$ increases when negative top gate voltages are progressively applied. This behavior shows that the channel material becomes *p*-doped during the fabrication process and that it presents a bandgap. Note that FETs having a graphene monolayer as a channel cannot be driven in the OFF state since this material has no bandgap. The existence of a bandgap in graphene is explained by the combined effect of the bandgap induced by HfZrO [16] and that due to nanopatterning (see [17] and the references therein), both effects inducing, a bandgap *E_g_* of about 0.18 eV. Assuming the device is OFF at $V_{TG}$ = 6 V and in the ON state at $V_{TG}$ = −6 V, at which the current is about 0.8 mA at $V_{D}$ = 6 V, the bandgap can be estimated to be 230 meV using the formula $I_{D,ON}/I_{D,OFF}=\exp(E_{g}/k_{B}T)$. Thus, as expected, the bandgap increases when the above-mentioned effects work simultaneously (i.e., when the graphene channel is nanopatterned and placed above HfZrO).

The bandgap opening in graphene is one of the reasons why we used HfZrO as a substrate for graphene transfer, the other being the very low roughness of HfZrO deposited by ALD, which is around 0.2–0.3 nm [19]. This is much smaller than the corresponding parameter of SiO_2_, which ranges between 1 and 3 nm depending on the deposition technique [19], and comparable with that of h-BN, which is about 0.4–0.6 nm [20].

In Figure 3b, we have represented the $I_{D}$−$V_{TG}$ dependence when $V_{BG}$ = 0. These hysteretic curves are strongly dependent on the various gate voltage [$-V_{TG}$,$V_{TG}$] sweeps, which is behavior typical for a ferroelectric transistor. The memory window, defined as the width of the hysteresis at mid-height, increases with the sweeping range and attains a large value of about 5 V when $V_{TG}$ when is swept between −6 and 6 V.

**Figure 4:**
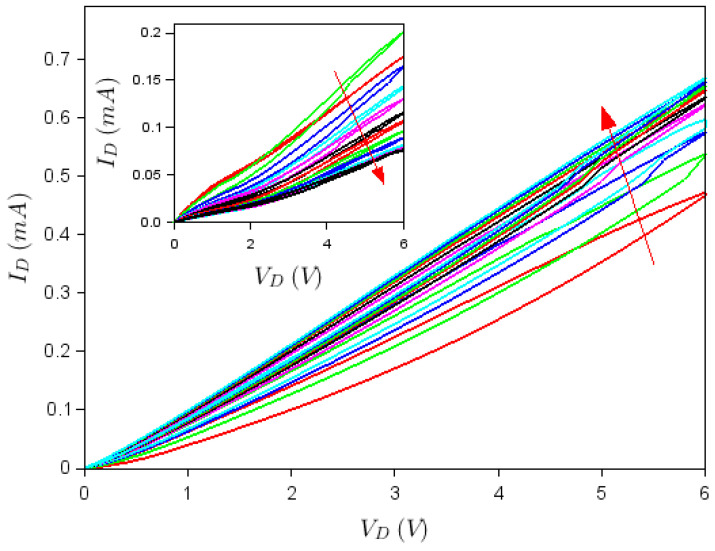
Dependence at $V_{TG}=$ −2 V, $V_{BG}$ = 0, and different drain voltage sweeps. The arrow indicates the direction of increase of the sweep number. Inset: same dependence for $V_{TG}=$3 V.

**Figure 5:**
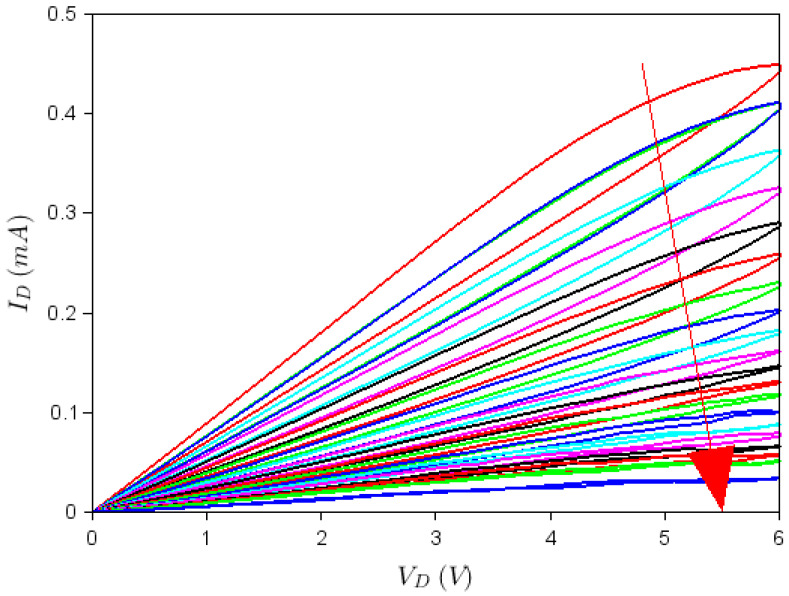
Dependence at $V_{TG}=-6$ V, $V_{BG}$ = 2 V, and different drain voltage sweeps. The arrow indicates the direction of increase of the sweep number.

Figure 4 and Figure 5 show that both top and back gates are able to rotate to a certain $I_{D}-V_{D}$ dependence by sweeping the drain voltage from negative to positive voltages and vice versa several times. More precisely, Figure 4 illustrates the $I_{D}-V_{D}$ dependence at successive sweeps of the drain voltage in the range [−6 V, +6 V], when $V_{TG}$ = −2 V and $V_{BG}$ = 0 (only the dependence for positive $V_{D}$ voltages is shown for clarity). As can be seen from this figure, the current increases with each sweep (the current variation from the first to the last, 10th sweep, is indicated by an arrow), with an apparent tendency towards attaining a steady state as the number of sweeps increases. This means that the conductance increases with time since the number of sweeps can be considered as discrete time steps. In our case, there were 30 s between two successive sweeps. On the contrary, the inset of Figure 4, which corresponds to $V_{TG}$ = 3 V and $V_{BG}$ = 0, shows that at similar successive sweeps of the drain voltage in the range [−6 V, +6 V], the current decreases if the top gate is positive until eventually a steady state is attained and the conductance decreases in time. These opposite behaviors are a consequence of the opposite polarities of the built-in field that develops in the graphene channel due to gate voltage values—a field that acts on charges trapped in the nanopatterned region of the graphene channel. Thus, the graphene/HfZrO/Al_2_O_3_/Si FETs act as memtransistors, memorizing not only the previous state but also indicating the top gate voltage polarity. Figure 4 illustrates that the change in drain current at various sweeps is significant, with the value of $I_{D}$ increasing 1.5 times at $V_{TG}$ = −2 V, while decreasing about 2.5 times at $V_{TG}$ = 3 V as the drain voltage is swept 10 and, correspondingly, 12 times. However, this significant change in $I_{D}$ at successive drain voltage sweeps in the range [−6 V, +6 V] can be enhanced even further (by about 12 times in 21 sweeps) for $V_{TG}=-6$ V and $V_{BG}$ = 2 V, as shown in Figure 5. For these values of the gate voltages, a built-in field develops in the graphene channel, which depletes the channel of charges trapped in the nanopatterned region repetitively at each drain voltage sweep, until the current through the FET is very small. Thus the current of memtransistors can be adjusted via the number of sweeps performed. Since, the drain current changes significantly during several sweeps, drain voltage sweeping can be seen as another method to change the ON/OFF ratio of the FET on-demand.

Finally, we measured two other types of graphene transistors fabricated in the same batch on identical ferroelectric substrates. First, we fabricated graphene/HfZrO FETs with a channel length of 1 µm, and they did not show memristor behavior. Additionally, in the same batch, we had transistors that were intentionally not nanopatterned and they did not show any memristive behavior. So, since the procedure of nanopatterning is also termed as defect engineering, it can now be said that a certain number of nanoholes (defects) are needed to trap enough charges to produce memristive effects.

All devices were measured at room temperature. The influence of the charge traps at the graphene/HfZrO interface can degrade transistor performance [20]. However, it was shown that HfZrO-based transistors have charge traps with low relaxation times, which do not influence the transistor performance at room temperature. The influence is more pronounced beyond 50 °C [21]. Moreover, it is well known that graphene monolayer electrical conductivity is not very sensitive to temperature as in the case of other semiconductors.

## 4. Conclusions

In conclusion, we demonstrated that nanopatterned graphene/HfZrO/Al_2_O_3_/Si FETs can act as memtransistors. The memtransistors were previously never fabricated at the wafer scale, and only on MoS_2_ flakes at a reduced scale. The combination of HfZrO and graphene monolayer is beneficial for graphene monolayer transfers since the average surface roughness of HfZrO, as measured with an AFM, was 0.2 nm—one order of magnitude lower than the roughness of SiO_2_.
